# Aerobiology and Its Role in the Transmission of Infectious Diseases

**DOI:** 10.1155/2013/493960

**Published:** 2013-01-13

**Authors:** Aaron Fernstrom, Michael Goldblatt

**Affiliations:** ^1^Mid-Atlantic Venture Investment Company, LLC, Washington, DC 20009, USA; ^2^Functional Genetics, Inc., Gaithersburg, MD 20818, USA

## Abstract

Aerobiology plays a fundamental role in the transmission of infectious diseases. As infectious disease and infection control practitioners continue employing contemporary techniques (e.g., computational fluid dynamics to study particle flow, polymerase chain reaction methodologies to quantify particle concentrations in various settings, and epidemiology to track the spread of disease), the central variables affecting the airborne transmission of pathogens are becoming better known. This paper reviews many of these aerobiological variables (e.g., particle size, particle type, the duration that particles can remain airborne, the distance that particles can travel, and meteorological and environmental factors), as well as the common origins of these infectious particles. We then review several real-world settings with known difficulties controlling the airborne transmission of infectious particles (e.g., office buildings, healthcare facilities, and commercial airplanes), while detailing the respective measures each of these industries is undertaking in its effort to ameliorate the transmission of airborne infectious diseases.

## 1. Introduction

Exposure to airborne pathogens is a common denominator of all human life [1]. With the improvement of research methods for studying airborne pathogens has come evidence indicating that microorganisms (e.g., viruses, bacteria, and fungal spores) from an infectious source may disperse over very great distances by air currents and ultimately be inhaled, ingested, or come into contact with individuals who have had no contact with the infectious source [2–5]. Airborne pathogens present a unique challenge in infectious disease and infection control, for a small percentage of infectious individuals appear to be responsible for disseminating the majority of infectious particles [6]. This paper begins by reviewing the crucial elements of aerobiology and physics that allow infectious particles to be transmitted via airborne and droplet means. Building on the basics of aerobiology, we then explore the common origins of droplet and airborne infections, as these are factors critical to understanding the epidemiology of diverse airborne pathogens. We then discuss several environmental considerations that influence the airborne transmission of disease, for these greatly impact particular environments in which airborne pathogens are commonly believed to be problematic. Finally, we discuss airborne pathogens in the context of several specific examples: healthcare facilities, office buildings, and travel and leisure settings (e.g., commercial airplanes, cruise ships, and hotels).

## 2. Aerobiology

Aerobiology is the study of the processes involved in the movement of microorganisms in the atmosphere from one geographical location to another [7], including the aerosolized transmission of disease. The aerosolized transmission of disease occurs through both “droplet” and “airborne” means. Droplet transmission is defined as the transmission of diseases by expelled particles that are likely to settle to a surface quickly, typically within three feet of the source [8–10]. Thus, for example, in order for an infection to be caused by droplet transmission, a susceptible individual must be close enough to the source of the infection (e.g., an infected individual) in order for the droplet (containing the infectious microorganism) to make contact with the susceptible individual's respiratory tract, eyes, mouth, nasal passages, and so forth [11]. In contrast, airborne transmission is defined as the transmission of infection by expelled particles that are comparatively smaller in size and thus can remain suspended in air for long periods of time. Airborne particles are particularly worrisome simply because they can remain suspended in the air for extended periods of time. Seminal studies from the 1930s and 1940s [8, 12, 13] demonstrated that airborne particles can remain airborne for as long as one week after initial aerosolization, and suggested further [13] that these particles likely remained airborne for much longer. They thus potentially expose a much higher number of susceptible individuals at a much greater distance from the source of infection [10, 11, 14, 15]. Depending on environmental factors (e.g., meteorological conditions outdoors and fluid dynamic effects and pressure differentials indoors), airborne particles are easily measured 20 m from their source [16]. These factors would be of no concern but for the fact that airborne bacterial, viral, and fungal particles are often infectious [17].

A complicating factor is the heterogeneous nature of droplet and airborne releases, which generally consist of mixtures of both single and multiple cells, spores, and viruses carried by both respiratory secretions and inert particles (e.g., dust) [17]. The origins of droplet or airborne infectious microorganisms are also heterogeneous: infectious particles may be generated from, for example, infectious persons, heating, ventilation, and air conditioning (HVAC) systems, and cooling tower water in hospitals [17]. All of these sources can produce airborne infectious particles [17]. Furthermore, *Aspergillus fumigatus* spores are common in dusts during outdoor and indoor construction, in air conditioners, ceiling tile, carpet, and other infectious aerosol carriers generated from dry sources; they may absorb water in the airborne state but still measure in the infectious particle size range [17]. Also, droplet and airborne transmission are not mutually exclusive. That is, independent of origin, particles carrying infectious microorganisms do not exclusively disperse by airborne or droplet transmission, but by both methods simultaneously [11].

Transmission of infectious disease by the airborne route is dependent on the interplay of several critical factors, primarily particle size (i.e., the diameter of the particle) and the extent of desiccation [17]. The literature suggests that a particle's size is of central importance in determining whether it becomes and remains airborne and infectious [18–23]. Simply illustrated, large particles fall out of the air and small particles remain airborne. The World Health Organization uses a particle diameter of 5 *μ*m to delineate between airborne (≤5 *μ*m) and droplet (>5 *μ*m) transmission [17, 24, 25]. How particle size affects spatial distribution in the human respiratory tract has been studied extensively. Some studies suggest that particles over 6 *μ*m tend to mainly deposit in the upper airway, while particles under 2 *μ*m deposit mainly in the alveolar region [26]. Other studies conclude that particles under 10 *μ*m can penetrate deeper into the respiratory tract, and particles over 10 *μ*m are more likely to deposit on the surfaces of the upper airways and are less likely to penetrate into the lower pulmonary region [27–35].

One of the challenges facing practitioners, particularly in an enclosed building, is that even large-sized droplets can remain suspended in air for long periods [17]. The reason is that droplets settle out of air onto a surface at a velocity dictated by their mass [17]. If the upward velocity of the air in which they circulate exceeds this velocity, they remain airborne. Hence, droplet aerosols up to 100 *μ*m diameter have been shown to remain suspended in air for prolonged periods when the velocity of air moving throughout a room exceeds the terminal settling velocity of the particle [17].

Another critical variable is the rate at which particles desiccate. Even large, moisture laden droplet particles desiccate rapidly. In his seminal paper, Wells showed that particles begin desiccating immediately upon expulsion into the air and do so rapidly: particles up to 50 *μ*m can desiccate completely within 0.5 seconds [8]. Rapid desiccation is a concern since the smaller and lighter the infectious particle, the longer it will remain airborne. Hence, even when infectious agents are expelled from the respiratory tract in a matrix of mucus and other secretions, causing large, heavy particles, rapid desiccation can lengthen the time they remain airborne (the dried residuals of these large aerosols, termed droplet nuclei, are typically 0.5–12 *μ*m in diameter [17]). Of further concern, very large aerosol particles may initially fall out of the air only to become airborne again once they have desiccated [17].

One reason why particle size is such an important variable in airborne and droplet disease transmission is that the ability of an infectious disease to cause an infection depends on the concentration of the microorganism, the human infectious dose, and the virulence of the organism [17]. Humans can acquire devastating infectious diseases through exposure to very low levels of infectious particles. For example, Influenza A is believed to transmit via airborne and droplet means, and the infectious dose of Influenza A for humans is very low [62]. Additionally, the infectious dose for *Francisella tularensis* is reported to be a single organism [17]. Only a few cells of *Mycobacterium tuberculosis *are required to overcome normal lung clearance and inactivation mechanisms in a susceptible host [17].

## 3. Common Origins of Droplet and Airborne Infections

The origins of infections resulting from droplet and airborne transmission are at the intersection of the clinical manifestation of disease, the site of infection, the presence of a pathogen, and the type of pathogen [11]. Thus, when investigating the origins of droplet and airborne infections, there are several well-known primary sources of infectious particles (see Table 1): vomiting, toilet flushing (i.e., toilet water aerosolization), sneezing, coughing, and talking. Moreover, toilet bowls, the water in them, and toilet seats may harbor infectious particles after the initial flush, making additional aerosolization of infectious particles possible with additional flushes for as long as 30 minutes after the initial flush [63]. Particle desiccation, discussed above, is important in this context. A single sneeze, for example, generates as many as 40,000 large droplet particles; most will desiccate immediately into small, infectious droplet nuclei [17], with 80% of the particles being smaller than 100 *μ*m [64].

The transmission of infectious diseases via airborne or droplet routes may also depend on the frequency of the initiating activity. For example, while a single sneeze may produce more total infectious particles than a cough [11, 28, 65, 66], Couch et al. reported that coughing is more frequent than sneezing during infection with Coxsackievirus A [67]. This finding suggests that coughing is a more likely method of airborne transmission for this disease than sneezing [67]. As coughing is also a common symptom of influenza infection [68, 69], it may also contribute to the airborne transmission of this pathogen.

Finally, infectious individuals are not always the immediate source of airborne infectious particles. Many people spend considerable time in office buildings, for example, and as a result become exposed to airborne pathogens that originate from nonhuman sources (e.g., molds, toxins produced by molds, pollen, pet dander, and pest droppings) [70–77]. The health effects associated with naturally occurring indoor biological air pollutants include disease, toxicoses, and hypersensitivity (i.e., allergic) diseases [70–77]. In addition, exposure to indoor biological air pollutants has been associated with “sick building syndrome,”a set of nonspecific symptoms that may includeupper-respiratory symptoms, headaches, fatigue, and rash and“appear to be linked to time spent in a building, but no specific illness or cause can be identified.” [78].

## 4. Environmental Considerations

While the airborne transmission of disease depends on several physical variables endemic to the infectious particle, environmental factors substantially influence the efficacy of airborne disease transmission. The environmental factors most often cited as modifying the airborne transmission of disease are temperature and relative humidity [17]. Together, they help determine whether or not an airborne particle can remain infectious [17]. For example, the size of infectious particles can change depending on relative humidity and temperature (i.e., factors that influence desiccation or hygroscopicity). An added complication is the fact that temperature and humidity influence viral, bacterial, and fungal particles differently [17].

Temperature is an important factor affecting virus survival [79, 80]. Generally, as temperature rises, virus survival decreases [79]. For example, low temperatures (i.e., 44.6°F–46.4°F) have been suggested to be ideal for airborne influenza survival, with survival decreasing progressively at moderate (i.e., 68.9°F–75.2°F) and high temperatures (i.e., >86°F). This relationship holds across a range of relative humidities (i.e., 23%–81%) [81]. Influenza has also been shown to be transmissible via airborne vector under cold, dry conditions [82]. While relative humidity is recognized to be a factor in the viability of airborne and droplet viral transmissions [79, 80], the exact relationship is presently not well understood. For example, the report of Arundel et al. that minimal survival for both lipid-enveloped and non-lipid-enveloped viruses occurs at relative humidities between 40% and 70% [82] contrasts with that for influenza noted above.

In general, bacteria are more resistant to temperature than viruses [83, 84]. Temperatures above 75.2°F are required to reduce airborne bacterial survival [83, 84]. This temperature relationship has been found with gram-negative, gram-positive, and intracellular bacteria: *Pseudomonas* sp. [83, 84], *Pasteurella* sp. [85], *Salmonella* sp. [86], *Serratia *sp. [87], *Escherichia* sp. [87–89], *Bacillus *sp. [87], *Bordetella *sp. [90], *Chlamydia* sp. [91], and *Mycoplasma *sp. [92]. The survival of aerosolized gram-negative bacteria (including *Pseudomonas* sp., *Enterobacter* sp., and *Klebsiella *sp.) has been reported to be greatest at high relative humidity and low temperature [93]. However, available data on the effects of relative humidity on the survival of airborne bacteria are thus far inconsistent. For example, airborne gram-negative bacteria (e.g., *E. coli*, *Salmonella *sp., etc.) are reported not to survive well at increased relative humidity [94, 95], while some airborne gram-positive bacteria (*Staphylococcus albus, Streptococcus haemolyticus, Bacillus subtilis, *and* Streptococcus pneumoniae* (type 1)) survive poorly at intermediate relative humidities [94–96]. Determining the rates of survival of airborne bacteria appears to be more complicated than with viruses [97, 98]. Even bacteria within the same structural classification (e.g., gram-negative) may vary in how they respond to different changes in temperature and relative humidity [79].

For fungi, extensive studies have characterized the levels of both indoor and outdoor airborne fungi and their spores [99, 100]. More than viruses or bacteria, airborne fungi and their spores have been suggested to have the potential to enter a building that uses natural ventilation. Certain species (e.g., *Aspergillus* sp.) are also well-known, potentially life-threatening airborne contaminants when introduced to immunocompromised patients (such as in a healthcare facility) [99]. Other fungi hazardous to the immunocompromised include *Blastomyces *sp., *Coccidioides *sp., *Cryptococcus* sp., and *Histoplasma* sp. [100]. Even in healthy people, individuals working consistently in indoor environments (such as an office or school) have shown hypersensitivity reactions such as rhinitis, sinusitis, or asthma in response to fungi exposure [79]. Relatively few laboratory studies have examined the airborne transmission of fungi and their spores in relation to temperature and relative humidity. Most data relating these variables to airborne fungi viability have been obtained in their natural environments [79]. Nonetheless, the results of such studies suggest a seasonal variation in airborne fungal and spore concentrations associated with common environmental conditions, including ambient temperature, relative humidity, precipitation, and wind speed [97, 98, 101]. Generally, fungi and their spores appear to be more resilient than viruses and bacteria, being able to withstand greater stresses due to dehydration and rehydration, as well as UV radiation [97, 98, 101].

Given the diversity of viruses and bacteria that can spread via airborne or droplet means (see Table 2), an understanding of aerobiology, typical origins of droplet and airborne infections, and how different environmental factors affect airborne and droplet particles is critical to any discussion of the amelioration or mitigation of infectious airborne and droplet particle transmission.

There are two principal challenges when working to ameliorate or mitigate the airborne transmission of infectious particles indoors: preventing infiltration and preventing transmission. We discuss the first in the context of office buildings and the latter in the context of healthcare facilities (both below).

## 5. Airborne Pathogens in an Office Building Setting

The principal approach to limiting airborne pathogens in an office building setting is the prevention of pathogen introduction (i.e., preventing infiltration) [102, 103]. Occupants of office and commercial buildings are exposed to airborne particles of all kinds. Routes of infiltration include the building's occupants, who unintentionally introduce airborne infections they harbor, the intentional introduction of dangerous biological agents, and the accidental entrance of viruses, bacteria, allergens, and molds (e.g., through an open door or window) [102, 103]. While buildings can be commissioned or recommissioned for configuration so that their occupants have reduced or limited exposure to airborne particles, many commercial buildings are not so configured or maintained [103]. As a result, the majority of people in high occupancy buildings are continually exposed to infectious microorganisms [103].

The current common denominator affecting the transmission and/or reduction of transmission of airborne particles in a building is its HVAC system. HVAC systems are intended to provide for the health, comfort, and safety of occupants by maintaining thermal and air quality conditions that are acceptable to the occupants [104, 105] through energy-efficient and cost-effective methods under normal conditions [106]. And, to the extent possible, they are expected to be responsive to hazardous exposures under extraordinary conditions [107]. A typical HVAC system has three basic components: (1) outdoor air intake and air exhaust ducts and controls, (2) air handling units (i.e., systems of fans, heating and cooling coils, air filters, and controls), and (3) air distribution systems (i.e., air ducts, diffusers and controls, return and exhaust air collectors, grilles, and registers, return and exhaust air ducts and plenums) [108]. HVAC systems perform multiple functions simultaneously, including controlling three known central variables in the airborne transmission of infectious particles: temperature, relative humidity, and air currents.

The introduction of airborne infectious agents into an office or commercial building varies with the microorganism [70]. Bacteria, molds, and allergens can easily enter a building through an HVAC air intake, spreading throughoutviathe air-handling system [102]. Building materials, carpets, clothing, food, pets, and pests are also known sources of introduction of airborne particles into an office or commercial building [102]. Molds and fungi represent an additional challenge, as theycan grow in damp or wet places (e.g., cooling coils, humidifiers, condensate pans, and filters) andthen serve as a continued source of contamination throughout the building. Bacteria and mold speciesare also known to grow in places where water has collected (e.g., ceiling tiles, carpeting, and insulation), and serve as a continuing source of contamination [102]. Viruses that are spread easily via airborne transmission (e.g., Influenza A) can be broughtinto a building byinfected individuals and potentially enter the return air system and be spread throughout a building by the HVAC system [102]. Such infected individuals may show no symptoms and thus hamper infection control measures (e.g., 30%–50% of humans infected with Influenza A show no symptoms [63]). In general, however, it should be noted that the extent to which HVAC systems contribute to the airborne transmission of disease has not been quantified [102].

A working group from the UPMC Biosecurity Center (Baltimore, MD, USA), including experts in air filtration, building ventilation and pressurization, air conditioning and air distribution, biosecurity, building design and operation, building decontamination and restoration, economics, medicine, public health, and public policy, concluded in 2005 that there are seven actionable items building owners and operators can undertake to immediately reduce the risk of building occupants to airborne particles [103, 109]. They are (1) to minimize filter bypass by sealing, caulking, and gasket filter cartridges, retainer banks, and tracking, (2) to commission buildings during design and construction, and recommission routinely to ensure that ventilation systems are operating as intended, (3) to increase air filtration to the maximumeconomicallyjustifiableMERV (Minimum Efficiency Reporting Value, a rating of air filter effectiveness) level, (4) to maintain filter systems by conducting regular inspections, (5) to ensure that HVAC maintenance staff has appropriate training to operate and maintain the HVAC system, (6) when economically feasible, tighten the building envelope to reduce the infiltration rate, and (7) when economically feasible, pressurize the building to reduce the infiltration rate. 

## 6. Airborne Pathogens in a Healthcare Facility Setting

While healthcare facilities are subject to the same infectious challenges common to all office and commercial buildings, they face an additional, unique challenge: high density populations of potentially contagious and immunocompromised people. This fact presents a unique challenge regarding infection control, as all respiratory pathogens can cause hospital-acquired infections [60]. In hospitals especially, viruses and bacteria spread easily via airborne transmission [62].

While recommendations for hospital hygiene include hand, instrument, and surface hygiene, even outstanding hygiene protocols for these vectors do nothing to stem the transmission of infectious airborne particles [63]. Not surprisingly, hospital-acquired infections have become ubiquitous [110], and healthcare facilities are now a common source for highly drug-resistant pathogens [111]. Adding to the problem is the fact that global public health leadership believes we are entering a “post-antibiotic era,” where once easily treated infectious diseases will become very difficult to treat [111].

Large quantities of infectious airborne particles are expelled during many routine patient bodily functions (see Table 1) endemic to healthcare facilities, and viruses and bacteria that can spread via airborne or droplet means are diverse (see Table 2). Many airborne microorganisms in healthcare facilities are increasingly found to have developed strong drug resistance [112]. The quantity and variety of hospital-acquired infections are also rising (see Table 3).

In the hospital setting, airborne infectious particles can have varied compositions. They can be single bacterial cells or spores, fungal spores, or viruses. They can be aggregates of several cells, spores, or viruses. They can also be biologic material carried by other nonbiologic particles (e.g., dust) [113]. Additionally, airborne infectious particles in hospitals span a wide range of sizes. Bacterial cells and spores range from 0.3 to 10 *μ*m in diameter. Fungal spores range from 2.0 to 5.0 *μ*m. Viruses range from 0.02 to 0.30 *μ*m in diameter [114]. Most infectious particles generated from human respiratory sources occur primarily as droplet nuclei, with a diameter of 0.5–5.0 *μ*m [114], allowing them to remain airborne—and highly infectious—for extended periods of time [17]. Influenza A illustrates the difficulty hospitals have containing highly infectious airborne particles that remain airborne and infectious for prolonged periods. Influenza A causes disease primarily in the lungs [63], so sterile hands, instruments, and equipment cannot prevent an infectious person from transmitting, or a susceptible individual from acquiring, the virus. And since 30%–50% of those infected with Influenza A are asymptomatic [63], it is often unknown when an infectious person is present. Furthermore, in public areas like emergency rooms, over 50% of detectable Influenza A viral particles are aerosolized [62]. Because the human infectious dose of this virus is very low [62], it is thus easy for individuals to become infected in such an environment.

Even with a known list of pathogens that can be transmitted from person to person in a hospital [60, 61] and evidence that various hospital-acquired infections are airborne transmission related [60], the extent to which airborne transmission contributes to the overall infection rate in hospitals continues to be debated [115, 116]. A source of uncertainty is no doubt the variability in the reported proportion of hospital-acquired infections resulting from airborne transmission. For example, Brachman estimated that airborne transmission was responsible for 10%–20% of all endemic hospital-acquired infections [117], while Kundsin concluded that airborne transmission accounted for 20%–24% of post operative wound infections [118]. Kowalski concluded that approximately one-third of all hospital-acquired infections involve airborne transmission at some point between the origin and the susceptible host [60]. Without conclusive evidence of the extent to which airborne transmission contributes to total hospital-acquired infections, healthcare facilities will continue to have a difficult task quantifying their facility-specific risk of airborne transmission and thus remain tentative in investing to ameliorate it.

**Figure 1:**
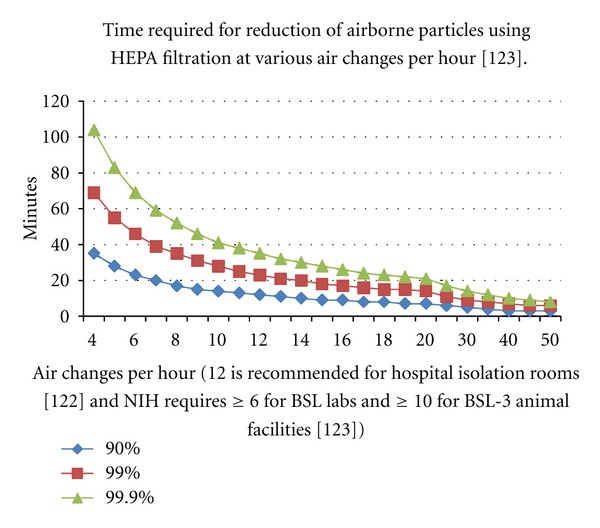


Healthcare facilities are subject to regulations and requirements relating to their HVAC systems [119]. Based on these regulations, hospitals currently attempt to reduce the airborne infectious disease load by (1) increasing the air changes per hour (a measure of how many times the air within a defined space is replaced per hour) in areas known to be problematic [120] and (2) utilizing different ventilation configurations and systems in specific areas (e.g., operating rooms, patient rooms, etc.) [121]. However, increasing the number of air changes per hour alone does not solve the problem. While the concentration of airborne infectious particles falls with increased air changes per hour, even very frequent air changing (within reason—it would be difficult to have a patient feel comfortable in a room where the air changed completely once a minute) does not radically reduce the airborne infectious particle count [120]. For example, Figure 1 plots the time it takes to reduce the airborne particle load in an air mass (ordinate) in relation to the number of air changes each hour passing through a high-efficiency particulate air (HEPA) filter (abscissa). Although developed in the 1940s, HEPA filtration is still considered the best-in-class method for removing infectious particles from air. The colored lines on the graph present data for removing 90% (i.e., 1 log reduction), 99% (i.e., 2 log reduction), and 99.9% (i.e., 3 log reduction) of the airborne particles. At 12 air changes/hour, which is the recommended minimum for hospital isolation rooms [122], it would take about 12 minutes to reduce the airborne particle load of a volume of air by 90% (blue), 23 minutes to reduce the load by 99% (red), and about 35 minutes to reduce the load by 99.9% (green). Note that these times are not markedly shortened up to 20 air changes per hour; recommendations for hospital isolation rooms is 12 [122], and NIH requires ≥6 for BSL labs and ≥10 for BSL-3 animal facilities [123]. Removing 90% or more of infectious particles from the air may be helpful, but not sufficient to eliminate airborne transmission of infection, particularly for viruses and bacteria that are extremely virulent and infect at very low exposure doses (e.g., Influenza A, *Francisella tularensis*, and *Mycobacterium tuberculosis*) [17, 62]. It should also be noted that the data in Figure 1 assume perfect mixing of air, which is known not to occur in practice.

Studying different ventilation configurations in specific areas, such as an operating room, using computational fluid dynamic modeling, reveals that airborne infectious particles spread throughout the space evenly and quickly no matter the configuration [121]. The American Society of Heating, Refrigerating, and Air Conditioning Engineers (ASHRAE) ran three different computational fluid dynamic models: (1) a conventional system with 1,500 cfm (cubic feet/minute) air flow and a conventional supply and exhaust, (2) a low supply, high-exhaust system, with 1,500 cfm and a conventional supply and exhaust, and (3) nonaspirating diffusers with 2,000 cfm, and a non-aspirating supply and conventional exhaust. The modeling in all three cases revealed that airborne particles spread throughout a space evenly and quickly regardless of HVAC configuration.

International guidelines and recommendations for airborne infection control have been issued by both the US Center for Disease Control and the World Health Organization for both resource-rich and resource-limited facilities [124–127]. The recommendations are based on a three-pronged approach to controlling airborne infections: administrative, environmental and personal protection [125–128]. While specific administrative controls differ according to setting, in resource-rich settings, persons suspected of having infectious respiratory diseases and patients who have received diagnoses of infectious respiratory diseases are to be placed in individual isolation. Quickly identifying and discriminating those that have active infectious respiratory diseases has been suggested as another effective method for controlling airborne infection [129]. In resource-limited settings, relocation to well-ventilated areas and application of cough hygiene protocols are recommended [130]. Indeed, simple natural ventilation has been shown to be a very useful approach to combating tuberculosis (TB) transmission in healthcare settings [131]. A recent study investigated rates of fresh air exchange achievable by natural means in health care settings [131]. More than 70 clinical rooms containing patients suffering from TB were studied (including emergency departments and outpatient clinics). Simply opening windows and doors provided between 28 and 40 air changes per hour, drastically reducing the amount of airborne infectious particles in the room [131].

Regarding environmental controls, several strategies are available to reduce exposure to infectious particles, including natural ventilation, mechanical ventilation, and upper-room ultraviolet light [132–135]. Mechanical ventilation delivering negative pressure and 12 air changes per hour is the standard of care for respiratory TB isolation [128], but these systems require delicate design and have high costs associated with installation. They also require ongoing maintenance, necessitating both resources and expertise. Unfortunately, poorly maintained mechanical ventilation systems have been widely documented in resource-rich settings [136, 137] and implicated in several TB outbreaks [124, 138–140].

Concerning personal controls, an essential personal protective practice is the regular and proper wearing of N95 respirator masks. The FDA defines an N95 respirator mask as “a respiratory protective device designed to achieve a very close facial fit and very efficient filtration of airborne particles. In addition to blocking splashes, sprays and large droplets, the respirator is also designed to prevent the wearer from breathing in very small particles that may be in the air.” While the ultimate effectiveness of these respirator masks is debated [124], respirator masks are believed to be the best currently available method of guarding against inhalation of highly infectious airborne particles such as tuberculosis [130].

Finally, as many microorganisms are susceptible to ultraviolet radiation, the use of upper-room UV fixtures has been widely studied. Given adequate room air mixing, infectious particles produced by in-room patients are likely to pass through the UV field (and possibly be sterilized) [130]. Escombe et al. [134] demonstrated that, provided that there is a sufficient circulation inside the room to mix the air, upper-room UV fixtures have been shown to be an effective intervention for use in infection control in high-risk clinical settings (e.g., tuberculosis). Nardell et al. [135] demonstrated as well that careful application of upper-room UV fixtures can be achieved without increasing the incidence of the most common side effects of accidental UV overexposure (e.g., eye and skin injury).

## 7. Airborne Pathogens in a Travel/Leisure Setting

An enclosed passenger cabin of a commercial airplane is an environment conducive to the airborne spread of pathogens carried by passengers or crewmembers [141]. However, as the environmental control systems used in commercial aircraft appear to restrict the transmission of airborne pathogens, the perceived risk by the public of airborne transmission of infectious disease on an airplane appears to be greater than the actual risk [142]. Nevertheless, a finite risk exists of droplet and airborne disease transmission while traveling in a commercial airplane. While there are four routes for the spread of microorganisms aboard an aircraft (e.g., contact, airborne, common vehicle, and vector borne) [143, 144], large droplet and airborne transmissions are thought in all likelihood to represent the greatest risk for travelers. The high density of occupants and their close proximity to one another are believed to contribute to this risk [141]. In this context, the ubiquity of commercial airline travel (over 1 billion passengers travel by air annually and 50 million of these travel to the developing world [145, 146]) may thus promote the spread of airborne pathogens over great distances. 

More specifically, several studies suggest that the risk of disease transmission to otherwise healthy passengers in an aircraft cabin is higher when sitting within two rows of a contagious passenger for a flight of more than eight-hour duration [142, 147–153]. While the eight-hour flight threshold is associated primarily with tuberculosis studies, many findings involving other pathogens support the general notion that infectious diseases routinely transmitted via airborne and droplet routes are effectively transmitted in aircraft cabins [147–150, 153–155].

One of the most critical factors in airborne disease transmission on an aircraft is cabin ventilation (or the lack thereof) [47, 142, 147, 148, 150, 151, 156–160]. One air change per hour of well-mixed air in any space is thought to remove 63% of the airborne organisms in that space [159, 160]. Typically, modern commercial aircraft cabins experience 15–20 changes of air each hour [141]. Hence, proper ventilation on commercial aircraft helps to reduce the transmission of airborne infectious particles [141], and thus it is not surprising that increased ventilation, as well as the filtration of recirculated air through high-efficiency filters, has helped to reduce the spread of airborne pathogens on airplanes [142, 148–150, 161]. At the very least, the recirculation of cabin air is known not to be a risk factor for contracting upper respiratory track infections [161]. In contrast, airborne transmission becomes widespread in passenger cabins with no ventilation, as shown by an influenza outbreak when passengers were kept aboard a grounded aircraft with an inoperative ventilation system [47, 148–150]. On balance, ventilation thus appears to be an important determinant of airborne infection risk on airplanes, and efforts to improve ventilation would be expected to reduce it [161]. Regarding specific pathogens that have been associated with droplet and airborne transmission in aircraft cabins, tuberculosis [148, 151, 156, 162–164], SARS [152, 153, 165–167], influenza [168–170], meningococcal disease [155, 169, 170], and measles [171–173] have all been studied.

Hotels and cruise ships share the same concerns as an office building or aircraft cabin, as these venues have in common enclosed spaces with large, dense populations. They are thus susceptible to airborne and droplet transmission via any of the mechanisms described above [38, 174].

## 8. Airborne Pathogens in a Biodefense Setting

A discussion of airborne pathogens as they pertain to biological terrorism is a too substantial subtopic for the present paper. Briefly, the hazards posed by airborne pathogens associated with biological terrorism are well described. The US and former Soviet Union maintained massive biological weapons stockpiles during the Cold War [175]. The occurrence of “confirmed bioagent” cases with “high value targets” continues to the present [176] and appears to be increasing [176]. Of recent, confirmed cases, the source of the biological material was a “legitimate supplier,” most of the perpetrators acted alone, and the majority of perpetrators had no medical or scientific expertise [176]. These findings suggest that biological terrorism could be a threat to public health and deserves to be included in any national biosecurity strategy.

## 9. Conclusions

Aerobiology is now an active discipline, employing contemporary techniques including computational fluid dynamics to study airborne particle flow, polymerase chain reaction (PCR) methodologies to identify infectious agents and quantify airborne particle concentrations in various settings, and epidemiology to track the spread of disease. However, the knowledge base is still limited, and translation to practice is in its infancy. For example, while the identity and concentration of airborne infectious particles under some conditions can be determined, few studies have thus far translated this information to useable estimates of infection rates for particular airborne particle sizes and concentrations, airflow conditions, exposure intervals, and pathogen virulence (among other variables). Such information would be of great value in helping to reduce the airborne transmission of infectious particles in all settings.

Practitioners of all kinds agree that the airborne transmission of infectious disease is a problem. Just how big or urgent a problem, however, continues to be debated. For example, there is currently a wide range in the reported frequencies of airborne transmission in hospital-acquired infections (10–33%). A better understanding of the true contribution of airborne transmission to infection rates would allow hospital administrators to determine the degree to which they should commit resources to minimize this vector of disease transmission. The same issue applies to similar environmental contexts, such as office buildings, aircraft cabins, cruise ships and hotels.

Practitioners of, and those responsible for, infection control in all settings are currently forced to use suboptimal (for the purpose), dated technologies to attempt to contain and eliminate the transmission of airborne infections (e.g., HEPA filtration systems were developed in the 1940s). High efficiency air filtration systems can be expensive to operate and easily fall victim to leakage and bypass problems that compromise the overall effectiveness of the system. However, as there is a lack of industry standards for evaluating new technologies that attempt to solve the airborne particle transmission problem, high-efficiency filtration remains the most widely deployed technology for this purpose.

## Figures and Tables

**Table 1:** Droplet or airborne microorganisms released from various activities.

Activity	Approximate particle count	Units
Sneezing [36]	40,000	Per sneeze
Bowel evacuation [37]	20,000	Per event
Vomiting [38]	1,000	Per event
Coughing [36]	710	Per cough
Talking [36]	36	Per 100 words

**Table 2:** 

Pathogens transmitted via droplet means	Pathogens transmitted via airborne means
*Bordetella pertussis* [39]	*Mycobacterium tuberculosis* [40–43]
Influenza viruses [44]	Rubeola virus [45]
Adenoviruses [46]	Varicella zoster Virus [47]
Rhinoviruses [48]	Variola viruses [25]
*Mycoplasma pneumoniae *[49]	Influenza viruses [47, 50]
SARS-associated coronavirus [51–53]	Rhinoviruses [48]
*Streptococcus pyogenes *[54]	Norovirus [55]
*Neisseria meningitidis* [56–58]	Rotavirus [59]
Respiratory syncytial virus (RSV) [25]	*Aspergillus* sp. [25]
*S. aureus* [25]	

**Table 3:** Known hospital-acquired infections [60, 61].

Bacteria	Viruses	Fungi
Group A Streptococcus	Rhinoviruses	*Aspergillus* sp.
*Mycobacterium tuberculosis *	Influenza viruses	*Zygomycetes* sp.
*Pseudomonas aeruginosa *	Parainfluenza viruses	*Histoplasma capsulatum *
*Klebsiella pneumoniae *	SARS	*Cryptococcus neoformans *
*Serratia marcescens *	RSV	*Coccidioides immitis *
*Corynebacterium diphtheriae *	Adenoviruses	*Blastomyces dermatitidis *
*Burkholderia cenocepacia *	Varicella zoster	*Mucor plumbeus *
*Chlamydia pneumoniae *	Measles	*Pneumocystis carinii *
*Nocardia asteroids *	Rubella	*Rhizopus stolonifer *
*Nocardia brasiliensis *	Poxviruses	
*Alcaligenes *sp.	Enteroviruses	
*Burkholderia pseudomallei *		
*Cardiobacterium *sp.		
*Moraxella* sp.		
*Burkholderia mallei *		
*Staphylococcus aureus *		
*Neisseria meningitides *		
*Bordetella pertussis *		
*Pseudomonas* sp.		
*Acinetobacter* sp.		
*Legionellae *sp.		
*Clostridia* sp.		
